# Assessing the Association of Elevated Zonulin Concentration in Stool with Increased Intestinal Permeability in Active Professional Athletes

**DOI:** 10.3390/medicina55100710

**Published:** 2019-10-21

**Authors:** Maciej Hałasa, Dominika Maciejewska, Karina Ryterska, Magdalena Baśkiewicz-Hałasa, Krzysztof Safranow, Ewa Stachowska

**Affiliations:** 1 Department of Human Nutrition and Metabolomics, Pomeranian Medical University, 71-460 Szczecin, Poland; dmaciejewska.pum@gmail.com (D.M.); ryterska.karina@gmail.com (K.R.); ewastachowska.pum@gmail.com (E.S.); 2 Department of General Pathology, Pomeranian Medical University, 70-111 Szczecin, Poland; poziomka@pum.edu.pl; 3 Department of Biochemistry and Medical Chemistry, Pomeranian Medical University, 70-111 Szczecin, Poland; chrissaf@mp.pl

**Keywords:** intestinal permeability, zonulin in stool, athletic activity, zonulin dependent mechanisms

## Abstract

*Background and Objectives*: The causative factors or conditions leading to increased intestinal permeability (IIP) have only been partly elucidated, suggesting excessive zonulin release to be a key factor among them. Likewise, it is known that athletic activity predisposes individuals towards the development of IIP; however, little is understood about the nature of this phenomenon. We decided to test the actual coincidence between IIP and increased stool zonulin (ISZ) in actively training athletes. *Materials and Methods*: We compared intestinal permeability tested with lactulose/mannitol differential absorption (lactulose/mannitol (L/M) test) and zonulin concentration in stool in 20 professional athletes (PRO), 9 amateur athletes (AMA), and 9 non-athletes (CTR). *Results*: The results confirmed that professional athletic activity showed significant positive association with intestinal permeability. ISZ was observed exclusively in athletes (CTR vs. AMA vs. PRO, respectively, 0% vs. 22% vs. 55%), and its prevalence was significantly higher in PRO than CTR. When we divided the participants into four categories related to exceeding the upper reference limits for both tested parameters (ISZ + or − and IIP + or −), significant differences were found between CTR and PRO; however, no significant differences were found between CTR and AMA or AMA and PRO. *Conclusions*: Our trial confirmed previous findings that professional athletic activity predisposes individuals to IIP. We also demonstrated that although ISZ was associated with intense training, there was no statistically significant association between ISZ and IIP in the tested group of professional athletes, which suggests the existence of additional mechanisms causing IIP.

## 1. Introduction

In recent years, there has been a tremendous growth of interest in the role of the gut in immune system function [1,2,3]. This, in turn, has created the urge for better understanding of the gut’s physiology and the intestine-related mechanisms underlying development of normally or abnormally functioning immunity [4]. Among the most important pathologies of the immune system that are frequently associated with malfunction of the gut are hypersensitivity-based diseases, including autoimmunities and allergies. Some of these diseases have been recently linked with increased permeability of the mucosal barrier in the intestine [5,6].

Increased permeability of the gut allows particles larger than normally accepted (such as amino acids, simple sugars, monoacylglycerol, and fatty acids) to cross the epithelial lining of the gut’s mucosa through the paracellular pathway [7]. As opposed to the appropriately absorbed small molecules, these larger food particles possess antigenic properties, which in certain circumstances (most probably underlined by genetic susceptibility) may lead to faulty activation of the immune system located in the gut’s wall, resulting in development of Celiac disease and other pathologies [8].

In recent history, it was commonly understood that in physiology, the majority of the transepithelial transport in the gut goes through the transcellular pathway. The paracellular pathway mostly explained the mechanism behind the increased permeability in pathological conditions [9]. However, the discovery of zonulin and the gradually growing understanding of its role in physiology has created the opportunity to reformulate this paradigm [10]. Production and release of zonulin, a protein that relaxes the intestinal tight junctions allowing for the paracellular transport in the gut’s mucosa, is one of the major mechanisms regulating intestinal absorption [9,10]. Its normal activity allows for paracellular transport in the gut at the physiological level, whereas the abnormally high release of zonulin may lead to pathologically increased intestinal permeability. Among factors that may pathologically upregulate zonulin release, gluten ingestion seems to play a well-established role [9,10]. Among other causes of increased intestinal permeability, the important factors are nonsteroidal anti-inflammatory drugs (NSAID) use and extreme physical (i.e., athletic) activity [11,12].

Only some of the agents or factors known to increase permeability of the gut have been directly confirmed to act through triggering of increased zonulin release [13]. Moreover, apart from the zonulin system, other mechanisms are known to cause increased intestinal permeability (IIP), such as direct damage to the epithelium. Some published data suggested significant correlation between the increased zonulin level in the blood serum and IIP, as measured by differential sugar absorption test, but this usually referred to particular groups of subjects tested (i.e., patients with diabetes mellitus type 1) [14]. Therefore, these findings may not be sufficient to formulate a universal statement regarding correlation of zonulin concentration increase and IIP development.

Another issue to be discussed is whether testing the zonulin serum level, which is frequently applied while testing intestinal permeability, most accurately reflects the actual status of this protein release in the gut. Zonulin concentration in the blood may potentially be affected by increased release of this protein outside of the gut, as it is suggested to regulate epithelial and endothelial tight junctions, as well as in the lungs and possibly other locations [15].

In addition to the abovementioned dietary causes, increased permeability of the gut has also been described to result from increased physical strain, typically found among vigorously training athletes [12,16]. There is no certainty, however, as to what the mechanism of IIP is and if there are any additional factors (possibly dietary) other than the physical activity itself influencing high frequency of permeability increase in athletes.

To test the relationship between zonulin release and intestinal permeability status in a group of subjects exposed to the risk of increased intestinal barrier permeability development, we examined the group of athletes during their vigorous training period. The zonulin level in their stools, as well as the lactulose to mannitol ratio in the urine (differential sugar absorption—lactulose/mannitol (L/M) test), were measured to test the relationship between these parameters. We also attempted to correlate the zonulin level and intestinal permeability with a set of anthropometric features and parameters typically related to athletes’ lifestyles and training regimens.

## 2. Materials and Methods

Three groups of healthy volunteers, all of whom were male between 19 and 43 years of age (median age 26 years), were recruited from three different populations as follows: typical non-athletic physical activity (n = 9) as the control group (CTR), mediocre intensity sport activity (n = 9) as the amateur athletes group (AMA), and very intense sport activity (n = 20) as the professional athletes group (PRO). All groups were recruited randomly, with the amateur athletes being recruited out of a local American football team and the professional athletes being recruited out of a mixed martial arts (MMA) fighters group. Professional athletes were tested during the peak of their competition season.

All of the participants were asymptomatic, free of any acute diseases, reportedly not suffering from any chronic illnesses, and receiving no long-term medical treatment. Moreover, the participants from AMA and PRO groups were medically classified to be officially fit for intense athletic training. The exclusion criterion for the trial was undergoing any antibiotic or nonsteroidal anti-inflammatory treatment within a month before the trial.

Upon inclusion to the study, the anthropometric parameters of all participants were measured using the seca body composition analyzer. The intestinal permeability (L/M test) and stool zonulin levels were also tested.

### 2.1. Anthropometric Assessment

All volunteers included in the test were first examined with the seca medical Body Composition Analyzer (mBCA) 515 for their anthropometric parameters assessment, which included height, weight, skin fold thickness, arm, waist, and hip circumferences, and body composition (fat mass, fat-free mass, skeletal muscles mass, cellular and extracellular water content, as well as phase angle value). The anthropometric examination was performed in the morning (fasting). Based on the measured parameters, the appropriate indices were calculated, including body mass index (BMI), waist–hip ratio (WHR), and fat and nonfat indices.

### 2.2. Stool Zonulin Assay

The concentration of zonulin in stool was determined with a competitive enzyme-linked immunosorbent assay (ELISA) kit (Immundiagnostik AG, Bensheim, Germany), following the manufacturer’s protocol. Stool samples were collected 1 day before the mannitol/lactulose test. Assay plate wells were coated with polyclonal anti-zonulin antibody; zonulin in the samples was conjugated to biotinylated zonulin tracer and then immobilized on the plate. Absorbance was measured with a photometer at 450 nm [17].

### 2.3. Differential Sugar Absorption Test (L/M Test)

#### 2.3.1. The Test Principle

The test used depended on the difference in pathways of gut absorption of lactulose and mannitol [5,18]. Intercellular tight junction damage or relaxation leads to the increase of urinary excretion of lactulose (absorbed primarily paracellularly) in relation to mannitol (absorbed transcellularly). The differential sugar absorption (L/M) test can be regarded as a direct marker of intestinal permeability.

#### 2.3.2. Sugar Ingestion and Urine Collection

The test began at 08:00, following an 8 h fast. Each participant emptied their bladder and collected 100 mL of urine for the blank sample. Subsequently, they drank 500 mL of water solution containing 7.5 g lactulose and 2 g mannitol. Thereafter, the participants were allowed to eat without restriction, except for specified foods consisting of milk and dairy products, simple sugars, vitamin C, and mannitol. All urine passed was collected into one container for 6 h. The 400 μL aliquots of urine from the collection container and from the blank sample were used in the test.

The following sugar derivatization and gas chromatography procedures are described elsewhere [19].

#### 2.3.3. Reference Limits and Statistical Analysis

We established internal upper reference limits for the lactulose to mannitol ratio (L/M) < 0.035 and zonulin < 30 ng/mL, referring to previous studies and our own experience [17,19].

We performed the statistical analysis using Microsoft Excel 2010 and STATISTICA 12.5. The Shapiro–Wilk test was used to determine the normality of the distributions of experimental variables, and for variables that were not normally distributed, the Mann–Whitney U test was used. To assess between-group differences in frequency of positive test results, exact tests for contingency tables were employed, including Fisher’s exact test for 2 × 2 tables [20]. The Spearman’s rank correlation coefficient (R^2^) was used to analyze correlations between the two basic parameters (zonulin in stool concentration and the L/M test results) and anthropomorphic, dietetic, and lifestyle parameters. Differences with *p* < 0.05 were regarded as statistically significant and are shown in figures.

#### 2.3.4. Bioethical Approval

The trial was performed in accordance with the protocol conditions approved by the Pomeranian Medical University Bioethics Committee (KB-0012/05/16) issued on 25 January 2016.

## 3. Results

### 3.1. Correlation of Permeability Parameters with Physical Status Parameters

The association analysis of the zonulin concentration in stool or the intestinal permeability, as measured by the differential sugar absorption test (L/M test), along with all of the parameters we registered upon physical examination and anthropometric measurements, showed very few significant correlations. The statistically significant associations (*p* < 0.05) found in the PRO athletes group were positive correlations between zonulin concentration in stool and waist diameter (R^2^ = 0.467), as well as between zonulin and total body water (R^2^ = 0.484).

### 3.2. Zonulin Concentration in Stool

The results of zonulin measurements were expressed as the concentration of zonulin in stool in ng/mL. We established the upper reference limit at 30 ng/mL, treating this value and all of the above as reflecting the increased zonulin level. This is in general accordance with other authors and our previous experience [17,19]. Moreover, the control group of non-athletes presented no results above the upper reference level in this test.

**Figure 1:**
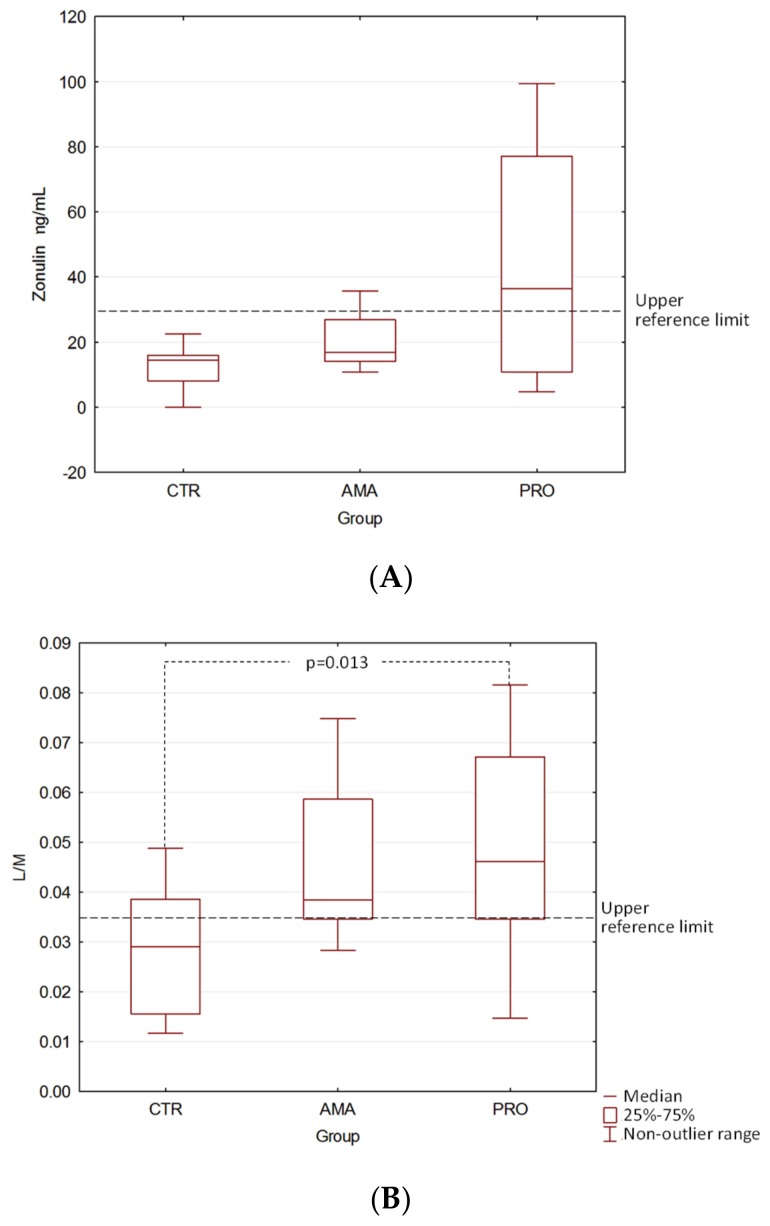
The results of the zonulin stool concentration test (**A**) and the differential sugar absorption test, expressed as the lactulose/mannitol (L/M) ratios (**B**) in the tested groups. The statistical significance was assessed with the Mann–Whitney U test.

**Figure 2:**
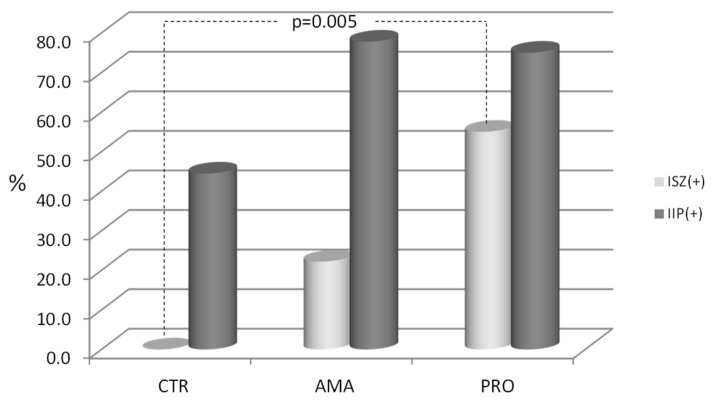
The proportions of the increased stool zonulin (ISZ) levels and increased intestinal permeability (IIP), as assessed with the L/M tests in all tested groups. The upper reference levels were established at 30 ng/mL for zonulin concentration in stool and at 0.035 for lactulose/mannitol ratio. Exact test was used to assess the differences between the groups.

The comparison of the zonulin stool concentrations between all groups showed no statistically significant differences (CTR vs. AMA *p* = 0.12, CTR vs. PRO *p* = 0.15, and AMA vs. PRO *p* = 0.26) (Figure 1A). After the stool zonulin concentration results had been transformed into the positive and negative results categories (increased stool zonulin (ISZ) + or −), the difference between the CTR and PRO groups was significant (*p* = 0.005); however, there was no significant difference between CTR and AMA (*p* = 0.47) or AMA and PRO (*p* = 0.13) groups (Figure 2).

### 3.3. Intestinal Permeability as Measured by Differential Sugar Absorption (L/M Test)

The results of the differential sugar absorption test for the examined groups are expressed as the ratio of lactulose to mannitol concentration in urine. Based on the literature, we established the upper reference level of the lactulose/mannitol ratio at 0.035, meaning that all results equal or above this value were treated as reflecting increased intestinal permeability [17].

When the results presented as lactulose/mannitol concentration ratios were compared between the control group and two tested groups, the difference turned out to be of significant magnitude between the CTR group and the PRO group (*p* = 0.013). Such a significant difference was not observed between the CTR and the AMA groups (*p* = 0.093) or between the AMA and PRO athletes groups (*p* = 0.759, Figure 1B). No significant differences were found when the groups were compared after transforming the raw lactulose/mannitol ratio results into the positive/negative results (IIP + or −) or categories of permeability status (CTR vs. AMA *p* = 0.34, CTR vs. PRO *p* = 0.21, and AMA vs. PRO *p* = 1.0) (Figure 2).

### 3.4. Zonulin Concentration in the Stool vs. Intestinal Permeability as Measured by Differential Sugar Absorption Tests

**Figure 3:**
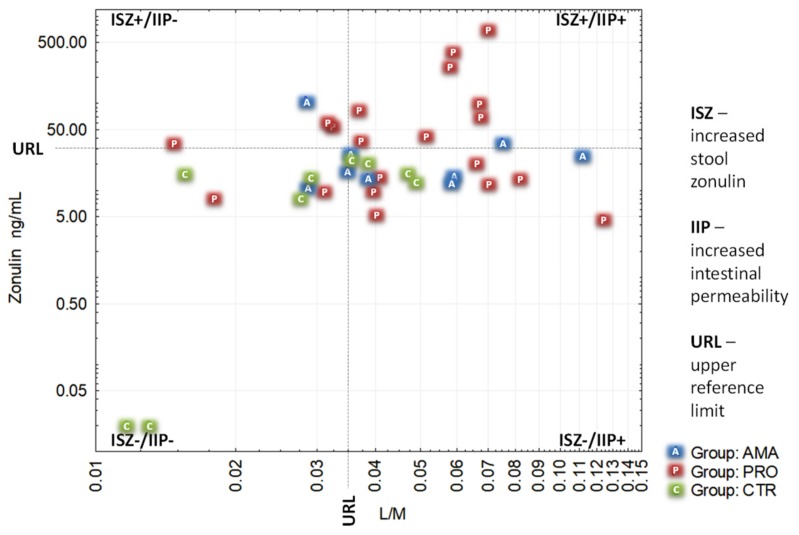
Two parameters (zonulin stool concentration and L/M ratio) based scattegram of participants distribution from three tested groups: non-athletes (CTR), amateur athletes (AMA), and professional athletes (PRO). Logarithmic scales were used for X and Y axes.

For clarity of presentation, we structured the results from all participants into 4 categories based on the upper reference limits established for both tests. These categories included all possible combinations of positive and negative results for zonulin stool concentration and L/M ratio (Figure 3).

**Figure 4:**
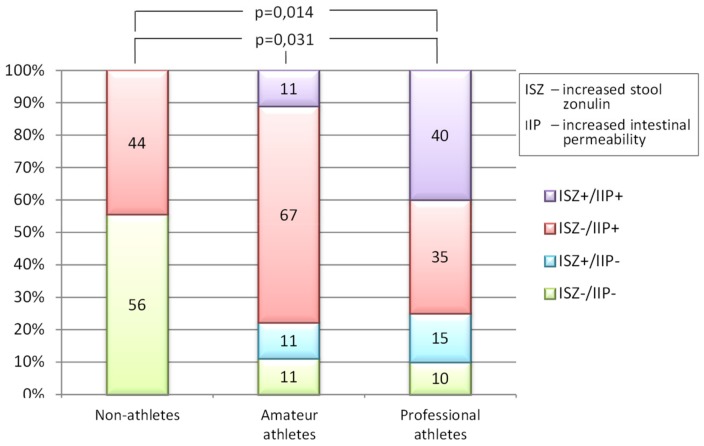
Categories of zonulin concentration in stool versus intestinal permeability, as found in each tested group. Increased stool zonulin (ISZ, + or −) was assessed by measuring the concentration of zonulin in stool and increased intestinal permeability (IIP, + or −) was assessed with lactulose/mannitol ratio test in urine. The categories are: ISZ (−) vs. IIP (+), ISZ (+) vs. IIP (+), ISZ (+) vs. IIP (−), and ISZ (−) vs. IIP(−). The difference in category distribution among groups was tested with exact tests, which showed statistically significant differences between all three (CTR, AMA, and PRO) groups (*p* = 0.031) and between the CTR and PRO groups (*p* = 0.014).

In this analysis, the statistically significant differences were found between 3 groups of participants (CTR, AMA and PRO) distributed across 4 categories of tests results (ISZ−/IIP+, ISZ+/IPP+, ISZ+/IPP−, and ISZ−/IPP−) (exact test for 4 × 3 table, *p* = 0.031). Further analysis showed that the difference was significant between the CTR and PRO groups (exact test for 4 × 2 table, *p* = 0.014) but not between CTR and AMA (*p* = 0.13) or AMA and PRO (*p* = 0.41) groups (Figure 4).

### 3.5. Associations between Tested Intestinal Permeability-Related Parameters in PRO Group

We found no statistically significant correlation between zonulin concentration in stool and intestinal permeability (expressed as L/M) in the PRO group (Rs = 0.105, *p* > 0.05). Similarly, no statistically significant association was found between ISZ (+/−) and IIP (+/−) categories in this group of athletes. We did not perform similar analyses in CTR or AMA groups due to the low number of subjects.

## 4. Discussion

The issue of increased permeability of the gut continues to grow as a major topic in various fields of medicine and related sciences. Sport medicine professionals have become interested in elucidating whether this abnormality affects the health status of training athletes and may eventually influence their performance. This has become especially important as it was discovered that malfunction of the intestinal barrier may develop in conjunction with intense exercise [12,16]. In addition, our previous experiments suggested that intense athletic exercise leads to an increase of permeability in the gut [18]. Therefore, recognizing the causes of increased permeability and the mechanism of its development is crucial in finding a solution to the problem.

Recent discoveries by Fasano placing zonulin in the center of the issue of IIP have brought hope that a remedy will be developed for the problem [13,21]. However, in view of the variety of known factors that may be at least partially responsible for IIP, it must be questioned if zonulin is always the key factor responsible for the opening of the paracellular absorption pathway. The most important reports pointing to zonulin in this role usually analyze the specific populations in which the nutritional or pharmaceutical factors seem to be responsible for permeability problems [6,7,8,9,13,21]. Our findings suggest that for increased permeability typical of actively exercising athletes, there is no association between permeability and stool zonulin concentration.

The results from this study confirm that the intense physical activity, as typically observed among professional athletes during their peak performance season, appears to be a factor influencing the development of increased intestinal permeability. This remains in accordance with the available literature [12,16,17,19]. In our study, this is expressed as elevation of at least one of the two tested parameters: differential sugars absorption (the L/M test) directly showing the existence of IIP, or the zonulin concentration in the stool test revealing one of the most important causes of IIP-increased zonulin release. The control group of people not involved in any intense physical activity had no increased stool zonulin (ISZ), but a substantial percentage of them presented with IIP. At least one of the two tested parameters seemed to be increased in a substantial proportion of tested athletes from amateur and professional groups (roughly in 90% of all athletes). While about three-quarters (75% and 78%) of athletes from both groups presented with IIP, ISZ was present in only 22% of AMA group and in 55% of PRO group athletes. Even more interestingly, IIP turned out to be coexisting with ISZ in 11% of amateur athletes and in 40% of professional athletes. Conversely, 67% of amateur and 35% of professional athletes presented with increased permeability without an increase in zonulin concentration. Although there was no statistically significant difference between the two athletes’ groups in this regard, our findings show that increased permeability of the intestinal barrier in sportsmen can coexist or not with increased zonulin release.

In view of the works by Fasano, to explain the lack of statistically significant association between ISZ and IPP in our study, it should be recalled that there are a variety of factors that can influence intestinal barrier permeability. Although not applicable to our study, as we excluded individuals currently using the NSAID, a good example of zonulin-independent increase of permeability may be the action of some agents triggering intestinal inflammation (i.e., NSAID). These medications can damage the intestinal barrier and thus cause IIP without involvement of zonulin in the process [11]. Even though the results we obtained from professional athletes (PRO) presented no association between IPP and ISZ, they stand in contrast in this regard to the non-athletes group, where all cases of IIP appeared without accompanying ISZ.

Another interesting and original finding from our study is that despite the common understanding coming from the discovery made by Fasano [7,10], the presence of ISZ does not necessarily coincide with IIP, as measured by the L/M test. This was observed in the PRO group (15% of all the tested athletes), as well as in the AMA group (11%). Currently, our knowledge is not adequate to satisfactorily explain this phenomenon. However, a possible reason could be that there is some unknown mechanism preserving the gut’s lining tightness, despite increased release of zonulin.

It is possible that the size of our tested populations prevented us from obtaining more statistically significant correlations between the intestinal permeability or stool zonulin concentration and the variety of anthropometric, lifestyle, and dietary parameters obtained through the questionnaire or physical examination. We found significant positive correlations between the stool zonulin level and waist diameter as well as total body water in intensely training athletes. The extent of this study does not permit us to form any profound conclusions from these findings, but it needs to be recalled that these body composition parameters may have some relationship with metabolic disturbances (possibly related to endocrine system problems), which have been demonstrated to coexist with increased zonulin level [22,23].

### Practical Applications

The long-recognized influence of intestinal homeostasis on a human’s overall health and performance and the fact that a large part of the population is involved in some kind of athletic activity have resulted in the need to test how intense physical activity may actually disrupt the digestive tract balance. Considering that appropriate permeability of the intestines largely defines proper intestinal function, and that this can potentially be distorted by intense athletic activity, it is important to find an accurate method to monitor this parameter in athletes.

Testing zonulin concentration has recently become the most attractive method for testing permeability status. Based on the results presented here, we suggest that at least in the case of actively training athletes, this may not best reflect the functional status of the intestinal epithelial barrier. Consequently, we suggest considering other methods, including the traditional differential sugar absorption tests (e.g., L/M test), for use in monitoring intestinal permeability status in athletes.

Although our study is of a rather preliminary nature, it presents some important observations that need to be confirmed in studies on a much larger scale. Additional studies could help to conclude whether there are any lifestyle, dietary, or sport activity factors or body composition characteristics that may influence the development of increased permeability of the gut. In addition, the issue of ISZ being found without accompanying IIP in some athletes needs to be elucidated, which may possibly be clarified by repeatedly testing a larger group of subjects to observe any possible dynamic correlation between ISZ and IIP.

## 5. Conclusions

- The extent of intestinal permeability, as measured by the L/M test, is significantly higher in professional athletes than in healthy non-athletes.
- Zonulin concentration in stool is elevated in professional athletes significantly more frequently than in healthy non-athletes.
- There is no association between ISZ and IIP in professional athletes.
- Stool zonulin concentration is positively correlated with waist diameter and total water body content in intensely training athletes.

The above permits us to state that zonulin seems to be just one factor among a possible array of factors leading to excessive intestinal permeability increase, and that elevation of its concentration in stool may not be obligatory for development of this abnormality.

## Abbreviations

ISZ	increased stool zonulin
IIP	increased intestinal permeability
CTR	control group
AMA	amateur sportsmen
PRO	professional athletes
L/M	lactulose to mannitol ratio
